# Exploring Innovative Approaches for the Analysis of Micro- and Nanoplastics: Breakthroughs in (Bio)Sensing Techniques

**DOI:** 10.3390/bios15010044

**Published:** 2025-01-13

**Authors:** Denise Margarita Rivera-Rivera, Gabriela Elizabeth Quintanilla-Villanueva, Donato Luna-Moreno, Araceli Sánchez-Álvarez, José Manuel Rodríguez-Delgado, Erika Iveth Cedillo-González, Garima Kaushik, Juan Francisco Villarreal-Chiu, Melissa Marlene Rodríguez-Delgado

**Affiliations:** 1 Universidad Autónoma de Nuevo León, Facultad de Ciencias Químicas, Av. Universidad S/N Ciudad Universitaria, San Nicolás de los Garza 66455, Nuevo León, Mexico; denise.rivera@unicepes.edu.mx; 2 Centro de Investigación en Biotecnología y Nanotecnología (CIByN), Facultad de Ciencias Químicas, Universidad Autónoma de Nuevo León, Parque de Investigación e Innovación Tecnológica, Km. 10 Autopista al Aeropuerto Internacional Mariano Escobedo, Apodaca 66629, Nuevo León, Mexico; 3 Centro de Investigaciones en Óptica AC, Div. de Fotónica, Loma del Bosque 115, Lomas del Campestre, León 37150, Guanajuato, Mexico; quintanillagabriela@cio.mx (G.E.Q.-V.); dluna@cio.mx (D.L.-M.); 4 Universidad Tecnológica de León, Electromecánica Industrial, Blvd. Universidad Tecnológica 225, Col. San Carlos, León 37670, Guanajuato, Mexico; asalvarez@utleon.edu.mx; 5 Tecnológico de Monterrey, School of Engineering and Sciences, Av. Eugenio Garza Sada Sur 2501, Col. Tecnológico, Monterrey 64849, Nuevo León, Mexico; jmrd@tec.mx; 6 Department of Engineering “Enzo Ferrari”, University of Modena and Reggio Emilia, Via P. Vivarelli 10/1, 41125 Modena, Italy; ecedillo@unimore.it; 7 Department of Environmental Science, School of Earth Sciences, Central University of Rajasthan, Ajmer 305817, Rajasthan, India; garimakaushik@curaj.ac.in

**Keywords:** microplastics, nanoplastics, biosensors, electrochemical sensor, photonic sensor

## Abstract

Plastic pollution, particularly from microplastics (MPs) and nanoplastics (NPs), has become a critical environmental and health concern due to their widespread distribution, persistence, and potential toxicity. MPs and NPs originate from primary sources, such as cosmetic microspheres or synthetic fibers, and secondary fragmentation of larger plastics through environmental degradation. These particles, typically less than 5 mm, are found globally, from deep seabeds to human tissues, and are known to adsorb and release harmful pollutants, exacerbating ecological and health risks. Effective detection and quantification of MPs and NPs are essential for understanding and mitigating their impacts. Current analytical methods include physical and chemical techniques. Physical methods, such as optical and electron microscopy, provide morphological details but often lack specificity and are time-intensive. Chemical analyses, such as Fourier transform infrared (FTIR) and Raman spectroscopy, offer molecular specificity but face challenges with smaller particle sizes and complex matrices. Thermal analytical methods, including pyrolysis gas chromatography–mass spectrometry (Py-GC-MS), provide compositional insights but are destructive and limited in morphological analysis. Emerging (bio)sensing technologies show promise in addressing these challenges. Electrochemical biosensors offer cost-effective, portable, and sensitive platforms, leveraging principles such as voltammetry and impedance to detect MPs and their adsorbed pollutants. Plasmonic techniques, including surface plasmon resonance (SPR) and surface-enhanced Raman spectroscopy (SERS), provide high sensitivity and specificity through nanostructure-enhanced detection. Fluorescent biosensors utilizing microbial or enzymatic elements enable the real-time monitoring of plastic degradation products, such as terephthalic acid from polyethylene terephthalate (PET). Advancements in these innovative approaches pave the way for more accurate, scalable, and environmentally compatible detection solutions, contributing to improved monitoring and remediation strategies. This review highlights the potential of biosensors as advanced analytical methods, including a section on prospects that address the challenges that could lead to significant advancements in environmental monitoring, highlighting the necessity of testing the new sensing developments under real conditions (composition/matrix of the samples), which are often overlooked, as well as the study of peptides as a novel recognition element in microplastic sensing.

## 1. Introduction

Plastic has emerged as one of the most widely used materials globally since its inception, thanks to its diverse properties, versatility, affordability, and countless applications. It is present in nearly every aspect of our daily life, appearing in a multitude of forms and presentations [1,2,3]. In 2022, global plastic production reached 400 million tons [4], with projections suggesting it may rise to 34 billion tons by 2050 [5]. However, this enormous production, coupled with widespread dispersal, inadequate waste management, and a slow degradation process [6], has led to the pervasive presence of small plastic particles across the globe, resulting in a variety of environmental and health challenges [7,8,9].

Small plastic particles can originate from primary sources, such as microsized components in cosmetics or hygiene or cleaning products, like exfoliants, toothpaste, and detergents. These components often include polyethylene (PE), nylon, polyethylene terephthalate (PET), and polypropylene (PP) microspheres [10]. Additionally, artificial fibers from the clothing and fishing industries contribute to primary sources of plastics when synthetic textiles, such as nylon, spandex, acrylics, and polyester, are washed. On the other hand, secondary sources refer to the unintended fragmentation of large plastic objects made of polystyrene (PS), polycarbonate (PC), polyvinyl chloride (PVC), poly(methyl methacrylate) (PMMA), PE, PP, and PET polymers into smaller particles due to environmental conditions. This fragmentation can occur from sunlight radiation, microbial biodegradation, or mechanical abrasion from external forces [11,12]. These tiny plastic particles are formally known as “microplastics” (MPs), a term introduced by Thompson et al. in 2004. MPs are defined as plastic fragments smaller than 5 mm; however, there are discrepancies within the scientific community regarding their size range, with definitions varying from 1 µm to less than 5 mm [13]. The term “nanoplastics”(NPs) typically refers to plastic particles measuring from 1 nm to less than 1 µm [14,15,16].

**Figure 1:**
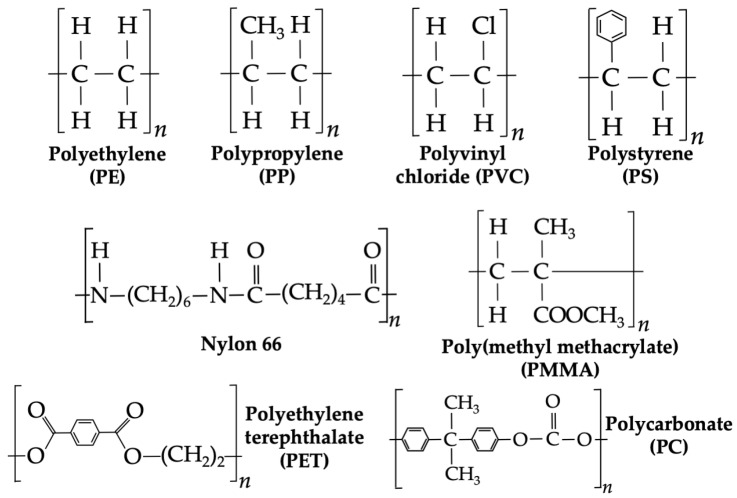
Chemical structure of the most common polymers present in MPs and NPs.

MPs and NPs are complex materials with a diverse range of physicochemical properties determined by their origin source (primary or secondary), environmental context, and varying degrees of environmental weathering. These microscopic plastic particles mainly comprise polymeric chemical structures (see Figure 1), conferring characteristic recalcitrance and hydrophobicity [17]. Secondary MPs and NPs originate from the fragmentation of larger plastic items, which often contain additives—such as plasticizers, pigments, light or thermal stabilizers, flame retardants, and antioxidants—designed to enhance their performance [18]. Their ongoing environmental transformation, particularly through weathering, significantly alters their physicochemical properties. Weathering processes modify the polymeric chemical structures, leading to oxidation and a shift towards a more hydrophilic nature. At the molecular level, weathering induces the formation of reaction byproducts, chain scission, molecular weight reduction, changes in chemical functionality, and alterations in the state of order within polymer chains, all of which contribute to the dynamic nature of MPs and NPs in the environment [19].

Due to their small size, both MPs and NPs are highly mobile [20,21] and have been found in various natural environments [22,23,24], from the deepest seabeds to the Himalayas [25,26,27,28], including populated areas [29,30]. These particles can be easily ingested, inhaled, or adsorbed by organisms, leading to their detection in numerous species, including plankton, birds, mammals, reptiles, and fish [31,32]. Their presence has also been reported in various food items, such as fresh fruits, vegetables, table salt, milk, beer, sugar, eggs, teabags, and bottled water [33,34,35,36,37,38,39]. Consequently, it is not surprising to find these microscopic particles in humans [40], where they have been detected in the lungs, liver, kidneys, placenta, blood, and feces [41,42].

Moreover, MPs and NPs are known for their ability to release harmful plastic monomers and additives/chemicals, including polybrominated diphenyl ethers (PBDEs), nonylphenol, bisphenol A, and phthalates [43,44,45]. They also possess a remarkable capacity to adsorb and accumulate various pollutants, such as polychlorinated biphenyls (PCBs), dichloro-diphenyl-trichloroethane (DDT), polycyclic aromatic hydrocarbons (PAHs), heavy metals, pesticides, and pharmaceuticals [46,47,48]. MPs and NPs can also serve as vehicles for microorganisms that attach to their surfaces [49,50,51].

As a result, the coexistence of MPs and NPs with these environmental pollutants poses a significant threat to human health, wildlife, and ecosystem integrity [52]. To effectively address MP and NP pollution, it is essential to develop accurate identification and quantification techniques that allow for the traceability of these particles across various matrices and the assessment of their removal [1,53].

This review presents an overview of current analytical methods for MPs and NPs and highlights the potential of innovative detection techniques for developing (bio)sensor platforms. These advancements aim to improve monitoring these particles and address the challenges and perspectives associated with future research in this area.

## 2. Current Methods for Analyzing Microplastics and Nanoplastics

**Figure 2:**
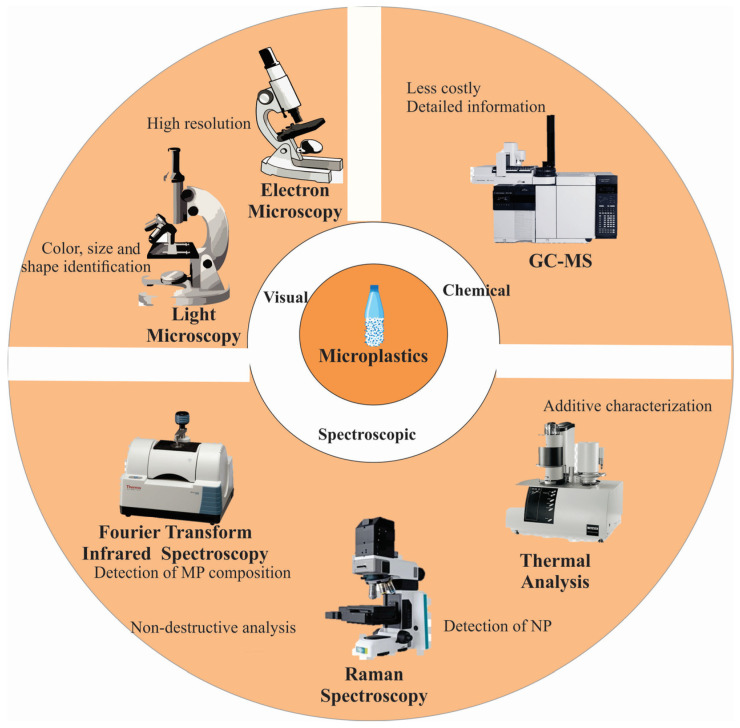
Scheme of the current methods for analyzing microplastics and nanoplastics.

MPs and NPs vary in size, shape, chemical structure, color, and the presence of additives such as colorants and plasticizers [54]. This diversity makes detection challenging, as each type of plastic has distinct physical and chemical properties [55,56]. MPs and NPs have been analyzed in various media, including water, sediment, sewage sludge, atmospheric deposition, and biota [57]. Although extensively studied across nearly all environmental compartments, the methodologies for detecting these particles differ based on the research objectives [58]. These methodologies are generally classified into three categories: physical analysis, chemical analysis, and spectroscopic analysis (see Figure 2).

The physical analysis of MPs and NPs typically involves visual identification using the naked eye or an optical microscope [59,60]. This method provides information about the particles’ size and shape, which can include forms such as pellets, fibers, beads, foams, sheets, and granules [61], as well as their color [62]. However, this approach is highly time-consuming and prone to misidentification, particularly as the size of the MPs decreases. Particles with a diameter of less than 1 mm are especially vulnerable to being overlooked or miscounted [63,64]. It has been reported that visual assessments by different operators can differ by as much as 30% [65]. Other techniques employed for this analysis comprise visual classification by size distribution analysis (such as laser diffraction) and scanning electron microscopy (SEM) [66].

Because physical characterization methods cannot distinguish MPs and NPs from other particles of similar size, chemical analysis is often conducted for more detailed detection. This includes vibration spectroscopy and thermal analytical methods [67,68]. Vibration spectroscopy is particularly useful for detecting and identifying polymers in environmental samples through their specific absorption spectra, without altering the composition of plastic particles [69]. The primary techniques used for this purpose are Raman spectroscopy [70,71] and Fourier transform infrared spectroscopy (FTIR) [11,72].

These methods are non-destructive, provide unique molecular fingerprints, and require only a minimal amount of sample [73]. However, their detection capabilities significantly decrease as the size of the plastic particles diminishes, making it challenging to analyze particles that are just a few micrometers or nanometers in size [74]. Additionally, the analysis can be time-consuming because spectra must be collected at each particle location [75].

Thermal analysis is considered a destructive method and encompasses various analytical techniques, including pyrolysis gas chromatography–mass spectrometry (py-GC-MS), thermogravimetry (TGA), and hyphenated techniques such as TGA–mass spectrometry (TGA-MS), TGA–thermal desorption–gas chromatography–mass spectrometry (TGA-TD-GC-MS), and TGA–differential scanning calorimetry (DSC) [73,76,77]. This approach is favored for its rapid analysis capabilities, high specificity, and adequate detection sensitivity, particularly in identifying the components of microplastics through the characteristic pyrolysis spectra of polymers [78].

However, while thermal analysis provides consistent detection results, it requires complex pretreatment and preconcentration of the samples. Some limitations include its destructive nature, the inability to determine the size, shape, and quantity of nanoplastics, and the potential influence of impurities in the samples on the results [79]. Table 1 displays some of the advantages and disadvantages of the current detection methods for MPs and NPs.

### 2.1. Visual Identification Methods

Visual inspection is the primary method for identifying the presence or absence of microplastics, either with the naked eye or under a microscope [59,71]. This method allows for the selection and classification of MPs, as well as the examination of attributes such as transparency, color, shape, and, size [47]. It can be applied in various environments, including air [80,81], the atmosphere [82], soils [83], water [84], wastewater from textile industries [85], wastewater from treatment plants [86], and sediment samples [87]. The most commonly used techniques for the visual identification of microplastics include stereo microscopy [88], scanning electron microscopy (SEM) [89], and fluorescence microscopy [77].

The stereomicroscope is the most affordable and accessible method for visual identification techniques [55,90]. Although this methodology can be quite time-consuming [77], visual separation is often considered an essential prerequisite [91] for effectively characterizing the main plastic-related aspects of environmental samples [2,92,93]. Stereomicroscopes are utilized for morphometric studies of microplastics [94]. Particles are classified visually based on their colors, shapes (such as fragments, fibers, and films), and sizes [20,95,96,97]. It is important to note that visual identification always carries a degree of subjectivity [98]. Although the precision of this method can be improved, it remains a lengthy process. Additionally, it does not allow for automation or differentiation between various types of polymers. Therefore, it is recommended to use other techniques for confirmation, as seen in studies conducted by [99,100,101,102,103].

Scanning electron microscopy (SEM) creates images by directing a beam of electrons at the surface of the sample [104]. As this beam sweeps over the sample, it causes the sample to re-emit photons and electrons [105]. The signals generated from the interactions between the sample and the electrons provide valuable information about the sample’s surface and composition [106]. SEM is known for producing high-resolution images of sample surfaces [107]. Its significant depth of field allows it to focus on a large area of the sample simultaneously, while also enabling detailed analyses of specific point locations. Sample preparation for SEM is relatively straightforward, as most SEMs require only that the samples be conductive [108]. The high clarity and excellent morphological detail offered by SEM help overcome the limitations associated with stereomicroscopes [109]. SEM analyses have provided insights into MP particles’ morphology and surface roughness [110]. The exceptional definition achieved with SEM allows for the distinction between synthetic MPs and other compounds, such as metals and minerals, that may cover the surfaces of the MP particles [110,111]. Although SEM has been widely used to identify MPs in numerous studies, it does have some drawbacks, including high equipment costs, time-consuming procedures, and limitations in color detection [88,104].

The filters (polarizers) used in polarized light microscopes allow light waves vibrating in a specific plane to pass through, enabling the investigation of the optical properties of samples. Polarized Light Microscopy is a valuable tool for analyzing and identifying polymers by examining their optical properties, such as birefringence and elongation signs [112,113]. This technique has successfully identified particles smaller than 20 μm [114]. In particular, Polarized Light Microscopy has identified particles of PET, PP, and polyethylene (PE) in water samples [115,116]. It has also been used to analyze samples from the digestive tracts of red mullet (*Mullus barbatus*) [117], as well as from wastewater treatment effluents and stormwater runoff [118]. However, to allow sufficient flow of polarized light, MPs must be very thin [119]. This means that thick and opaque MPs cannot be detected using this method [112], as the crystallinity of a plastic material affects how plane-polarized light behaves when it interacts with the plastics. Additionally, wood and paper can sometimes exhibit characteristics similar to microplastics under a polarized light microscope [116].

Fluorescence microscopy is a valuable tool for observing objects that emit fluorescent light, such as white and opaque plastics [120]. This technique has been effectively used to identify MPs in various matrices [121], including zooplankton samples [122], seashore sands [123], and air samples [124]. However, one significant limitation of this method is that it can be affected by interference from chemical additives, as well as microbiological, organic, or inorganic materials during the analysis [77,125].

### 2.2. Spectroscopy

Spectroscopy is an analytical technique that detects the absorption or emission of electromagnetic radiation by the sample being studied. In the analysis of microplastics, spectroscopy offers numerous advantages: it does not destroy the samples, requires only a small amount of material, provides better detection capabilities, and is environmentally friendly [68,126]. Among the various spectroscopic techniques, two have proven particularly effective for identifying and determining the chemical composition of MPs and NPs: Fourier transform infrared spectroscopy (FTIR) and Raman spectroscopy [127,128,129]. Each technique has its own advantages and disadvantages. When used together, FTIR and Raman spectroscopy provide complementary data that are more informative than using either technique individually. However, it is important to note that these techniques can only measure MPs and NPs that meet specific criteria: they must have a certain thickness, be transparent, and possess a regular morphology. If these conditions are not met, the signal may be compromised due to the reflection errors caused by light scattering [130].

The simplicity of its use makes FTIR a widely adopted method for analyzing and determining the structure of MPs [131]. This technique utilizes interferometry to encode information from a sample placed in an infrared beam. This information is then decoded through Fourier transformation, resulting in spectra that are used to identify or quantify MPs, as each type corresponds to a unique infrared spectrum [125,132,133]. Numerous studies have employed FTIR to identify and quantify MPs and NPs in various environments, including the atmosphere [109], soils [134], foods like milk [135] and table salt [136], as well as in organisms such as plankton [137] and marine fauna organs, including dolphin stomachs [138] and human blood [139]. It is also the most commonly used technique for characterizing MPs and NPs in major seafood groups [140,141]. One of the advantages of FTIR is its simple sample preparation, making it a more cost-effective option. Additionally, since the process is non-destructive, the sample remains intact during analysis [75]. FTIR can also assess particle aging by observing surface oxidation [142]. Using this method, MPs can be identified within a size range of 50 to 500 μm quickly, with a high spatial resolution of 10 to 20 μm [143]. Despite its high sensitivity, FTIR may struggle to detect NPs at low concentrations [144,145], as well as MPs and NPs that are close to or smaller than 10 μm [140]. Additionally, identifying a mixture of different polymers in extracted MPs can be challenging, even when compared to the existing FTIR reference spectral library [83,146]. While FTIR equipment is generally less expensive than other analytical techniques, costs can rise due to additional accessories and software [147].

Raman spectroscopy is a photonic technique that provides structural and chemical information about materials [148]. This analysis is based on measuring the light scattered by a material when it is illuminated by a monochromatic light beam [149]. A portion of this scattered light undergoes changes in frequency compared to the incident light, which enables the identification of the material [109]. Raman spectra are particularly useful for observing the main structures of specific polymers by characterizing their unique spectral fingerprints [79,150]. This capability has led to its increasing popularity in the detection of MPs in various matrices [126], such as atmospheric aerosols [151], organisms like commercial fish [99] and tropical sharks [152], animal organs such as mouse liver [153], and everyday cosmetics [110]. One of the key advantages of Raman spectroscopy is that it is a non-destructive technique, characterized by simplicity in sample preparation and fast, non-contact detection speed [154]. It can reveal information about the size of MPs in small ranges (1 μm), even at low concentrations [110,149,155]. Moreover, it provides insights into the chemical composition of MPs [156]. Since it is not influenced by the presence of water or humidity in environmental and biological samples, Raman spectroscopy is particularly well suited for detecting samples from aqueous environments [157,158]. Additionally, Raman systems are often portable, allowing testing to be conducted directly on site [74]. However, there are some disadvantages to using Raman spectroscopy for detecting MPs [159]. One significant limitation is that it often fails to provide quantitative information about the materials [156]. Furthermore, when analyzing small MPs, some characteristic Raman peaks may be submerged within the fluorescence signal, interfering with detection [160]. This issue can be exacerbated by certain plastic additives that lead to spectral distortion and a low signal-to-noise ratio in the Raman spectra of MPs [161,162,163]. Lastly, to obtain reliable results, a clean surface is necessary, which can be challenging when working with MPs found in environmental samples that may be coated with organic or inorganic compounds [164,165].

### 2.3. Thermoanalytical Methods

Thermoanalytical methods involve the decomposition of polymeric compounds at temperatures exceeding 500 °C, followed by the quantification of the resulting pyrolysis products [166]. These techniques are sensitive [167,168] and enable the accurate and uniform identification of various types of polymers [169]. They provide both qualitative and quantitative information [170,171] across the full range of MP sizes [172], including NPs [173,174]. One key advantage of these techniques is that they do not require any sample pretreatment [175] and can analyze minimal sample quantities, typically ranging from 5 to 200 mg [176]. Additionally, thermoanalytical methods allow for the simultaneous identification of organic products related to polymers [177] and additives and degradation byproducts [10]. They also demonstrate greater robustness against impurities [178]. However, there are limitations to these analytical methods, particularly in distinguishing between copolymer compounds [173,179]. Furthermore, due to the destructive nature of thermal analysis, morphological information such as size and shape cannot be obtained [174]. The primary thermal techniques used include pyrolysis coupled to gas chromatography–mass spectrometry (Pyr-GC-MS), thermal extraction desorption coupled to gas chromatography–mass spectrometry (TED-GC-MS), and differential scanning calorimetry (DSC).

In the Py-GC-MS technique, heat is used to break down polymers into lower molecular weight products, which can then be separated by gas chromatography and identified through mass spectrometry [166,180]. This technique has been employed to analyze MPs in various samples, including municipal wastewater treatment plants [181,182], as well as in organisms such as shellfish [183], seabass [184], cucumber plants [185], and tissues of aquatic animals [186]. It has also been used to study MPs in water [187], sediments [188], soil [189], rice [190], and even human blood [191]. Py-GC/MS offers several advantages, including the ability to analyze small sample sizes and a fully automated system [192,193]. This technique provides detailed insights into the chemical composition of plastic particles, as well as their endogenous additives and the environmental contaminants they absorb [165,194,195]. Additionally, it can detect NPs [166,174]. However, there are drawbacks to this method. Samples may contain various polymers even after pretreatment. When a mixture of polymers is pyrolyzed, reactions can occur between the resulting pyrolysates, potentially introducing systematic errors in the quantification of MPs [194,196]. Furthermore, data processing can be complex, and the limit of detection (LOD) can vary significantly. Because only minimal quantities are pyrolyzed (less than 0.5 mg), samples often need to be preconcentrated to achieve reliable LOD values [166,195]. The analysis of larger MPs is also limited by the size of the pyrolysis tube [168,193]. Lastly, it is important to note that samples are consumed and partially destroyed during the analysis [166].

The TED-GC/MS technique is a powerful analytical method that allows for the decomposition of molecules at low boiling temperatures, providing valuable structural information and a comprehensive understanding of changes occurring in polymers as temperature increases [172]. Following this decomposition, the resulting gaseous compounds are sorbed onto a solid adsorber. These adsorbed compounds are then thermally desorbed and identified using GC/MS [194,197]. This method is particularly effective for identifying polymers found in complex matrices, including drinking water supply systems [172], municipal wastewater [166], ferment residue samples [198], sediments [199], and for quantifying MPs in spiked suspended matter [175,200]. One major advantage of TED-GC/MS is its versatility; it can be applied to both organic and inorganic matrices with diverse types and concentrations of MPs [175]. The method is also rapid, with analyses of water and air filtrate samples typically taking between 2 to 3 h [201,202]. The use of a thermogravimetric analyzer further enhances its appeal by accommodating samples with higher masses, making it suitable for small-scale heterogeneous samples. It has achieved a remarkable limit of quantification of 135 pg for PS [198]. Additionally, TED-GC/MS can identify and quantify the mass content of MPs in environmental samples up to 100 mg based on the analysis of polymer-specific decomposition products. When a homogeneous sample of this size is available, no extensive preparation steps are usually required. This technique can analyze both the polymer and its additives in a single experiment [172]. However, there are several disadvantages to consider. The analysis can be costly, destructive, and time-consuming [203]. Sample preparation stages, including grinding and mixing, are necessary, and the method does not provide data related to the size and shape of the particles [204]. Furthermore, data analysis is intricate and can pose operational challenges [205].

A combination of physical and chemical analyses has enhanced the detection of MPs and NPs [206]. Researchers worldwide are actively working to improve these techniques or develop new, practical, and accurate methods focused on optimizing factors that can potentially affect the detection of these plastic particles. Such factors include organic matter, fluorescent dyes, and non-polar functional groups [207]. Some of these new technologies are still in the initial development stages and include semi-automatic innovative image processing, polarimetry, electrochemical impedance spectroscopy, plasmon-enhanced fluorescence, photoelectrocatalysis, and the analysis of MPs and NPs through attached pollutant particles [208,209]. The following section summarizes the novel analytical methods used to identify and quantify MPs and NPs, along with their advantages and limitations.

## 3. Biosensors for Microplastic Detection

In recent decades, traditional analytical tools have been used to detect MPs in various environmental matrices. Each of these tools has its own advantages and disadvantages. Introducing novel detection methods that rely on affordable and user-friendly instruments could create more efficient analytical devices [210]. Research focused on materials, procedures, and strategies for functional optimization across different areas has promoted the development of new sensing techniques [211,212,213,214,215]. Such sensing techniques encompass biosensors and autonomous integrated devices that detect analytes using biological materials connecting directly to a transduction component [216]. The most notable features of biosensors include stability, selectivity, sensitivity, high specificity, non-invasiveness, reproducibility, linearity, and the ability for remote sensing. These qualities support the introduction of high-potential analytical approaches [217]. However, challenges such as complexity, limitations in high-absorption samples, and surface interference may restrict their application [210].

Biosensors are composed of three main components: a bioreceptor, a transducer, and a reader device [218]. They can be classified based on the type of receptor employed in the detection, such as enzymes, proteins, oligonucleotides, antibodies, microorganisms, whole cells, tissues, or nanoparticles. In this sense, incorporating a (bio)receptor provides a higher specificity to sensing detection by interacting specifically with plastic molecules, even in the presence of contaminants or materials within the sample matrix [219]. Depending on the sensing approach, these contaminants (interferences) in the sample could also generate electrochemical or optical responses during the microplastic analysis, obtaining false positive results or overestimating concentrations. Also, receptor-based methods help avoid pretreatment steps (like filtration and sedimentation) commonly employed to eliminate these contaminants. In terms of advantages, it not only allows distinguishing microplastic particles from other materials in real scenarios (such as soil particles, vegetal debris, algae, bacteria, etc.) but also enables discriminating among the different types of plastic according to the polymeric nature of their chemical structure (i.e., polyethylene, polyethylene terephthalate, and polypropylene). Finally, receptor-based assays can increase the limit of detection of the sensor since the interaction between the receptor and plastics occurs at the molecular level (allowing low concentration detection) [219].

Additionally, biosensors can be categorized by the type of transducer used, which may be electrochemical, optical, thermal, etc. [217,220,221,222]. Optical biosensors are particularly promising due to their low detection limits, high sensitivity, and capacity for multiplexed detection. The most common types of optical biosensors, depending on the ligand–analyte pair, employ various detection methods, including surface-enhanced Raman spectroscopy (SERS), surface plasmon resonance (SPR), integrated microring resonators (MRRs), interferometers—especially Mach–Zehnder interferometers (MZIs)—fiber Bragg gratings (FBGs), and photonic crystal sensors of various types [223]. Currently, SPR biosensors are known to provide the best detection limits. Meanwhile, although they are less commonly used, interferometers and resonators also demonstrate significant potential due to their ability to reach low detection limits. Antibodies are often the preferred biological recognition elements because of their high affinity and durability [224,225].

The increasing interest in biosensors across various sectors can be attributed to recent research developments that have created inexpensive, highly effective devices with excellent sensitivity and specificity. These advancements enable biosensors to differentiate between target substances and potential interfering substances effectively [226]. As a result, biosensors have emerged as a novel technique with a wide range of applications, including clinical settings [227,228,229], pharmaceuticals [230,231,232], the agri-food sector [233,234,235], industrial uses [236,237,238], and environmental applications [238,239,240].

Given the threat microplastics pose to the environment and living organisms, biosensors are a powerful tool for detecting these pollutants [241]. Their high sensitivity enables the detection of low concentrations of analytes, providing a reliable response to changes in concentration [222]. Table 2 summarizes the analytical performance of the sensors employed in detecting microplastic particles and their related harmful compounds.

### 3.1. Electrochemical Sensing Approaches

**Figure 3:**
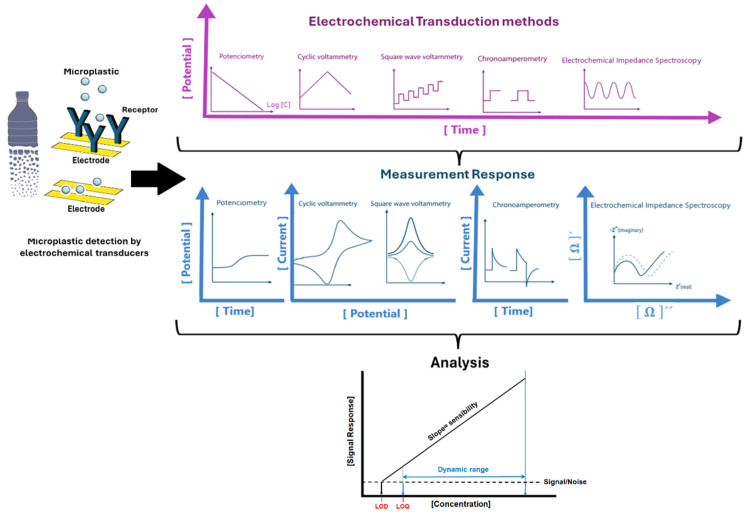
Scheme of electrochemical (bio)sensing approaches (with or without receptors) for the analysis of microplastics.

Electrochemical techniques enable both qualitative and quantitative detection of contaminants, including MPs and NPs. Based on the type of signal quantification, electrochemical sensors and biosensors can be categorized as voltammetric, amperometric, or impedimetric devices (see Figure 3). This classification depends on the electrical properties being measured, which include current, potential, resistance, or changes in impedance [271]. Significant research has been carried out on these methods due to their low production costs (specifically in electrode fabrication), portability, and speed of analysis. This advancement is evident in the increasing number of studies that report the detection of MPs using electrochemical approaches.

The differentiation of plastic particles from a mixed sample containing other particulate materials relies on their electronic properties. For instance, a resistive pulse sensor detects changes in current as particles pass through a restricted sensing area, producing different signals based on the size, shape, or concentration of the MPs [272]. The study by Pollard et al. (2020) established the use of resistive pulse sensors to screen MPs and algae, successfully differentiating between spherical and rod-shaped algae and the irregular structures of MPs released from tea bags. The presence of MPs in the teabag was quantified at a concentration of up to 6.52 × 10^−4^ particles/mL, with an average particle size of 21.9 µm. The sensor was able to detect particles ranging from 2 to 30 µm as the MPs passed through the sensing zone, even in salt concentrations ranging from 2.5 × 10^−4^ to 0.1 M [242]. However, it was observed that the electrochemical signal was influenced by the ionic strength of the electrolyte, as well as the porosity and shape of the MPs [242].

In terms of impedance spectroscopy sensors, the electrodes measure the impedance shift of the MPs moving through a medium. When measurements are taken at high frequencies, it is possible to differentiate the electrical properties of the particles. Conversely, at lower frequencies, the impedance is directly related to the volume or size of the particles [273]. Colson et al. (2021) reported using impedance to detect PE particles in tap water, with sizes ranging from 212 to 1000 µm, as well as seeds of the same size, which created some interferences. The impedance changes were measured at frequencies of 1.1 MHz and 10 kHz, resulting in a recovery rate of over 90% for MPs sized between 100 to 300 mm, with a mere 1% false-positive rate in identifying biological particles as MPs [243]. Additionally, PE spherical particles ranging from 1 to 22 µm have been characterized using chronoamperometry measurements with a three-electrode scheme, where the working electrode was made of carbon fiber wire. The particles themselves did not react during the measurements; rather, they acted as oxygen carriers. Their collision with the working electrode generated a change in current, observed as reductive spikes attributed to the reduction of oxygen to hydrogen peroxide [274]. The same method was also utilized for detecting PS-MPs. In this case, the dispersion of particles on carbon electrodes resulted in a blockage of the ferrocene mediator’s charge transfer. Consequently, the decrease in current (in the order of picoamperes) recorded during chronoamperometry was proportional to the diameter of the MPs, which ranged from 0.1 to 10 µm. The concentration of the particles was quantified within the range of 0.005 to 0.500 pm [244].

In a separate study, Davies and Crooks (2020) found that the polarization technique is an effective alternative method for detecting MPs. This technique leverages the electrophoretic mobilities of particles to sort them based on Faradaic ion concentration polarization, which influences their interaction with electric field gradients. Specifically, particles with high electrophoretic mobility tend to aggregate in areas of lower electric field strength, while particles with lower electrophoretic mobility concentrate in regions of higher electric fields [275]. Consequently, plastic particles can be sorted into different chambers within microfluidic systems according to their electrophoretic mobility, allowing them to be separated based on the trajectories determined by the electric field gradients created by the electrodes.

Recent research has highlighted the use of dual transduction principles in detecting MPs. For instance, Wang et al. (2022) designed a sensor that utilizes both impedimetric and voltammetric principles for detecting PE-MPs. This sensor features a three-electrode setup, consisting of a carbon working electrode, a titanium counter electrode, and a silver/silver chloride (Ag/AgCl) reference electrode. It incorporates an electroactive bacterium capable of producing an exoelectrogenic response. However, the microbial cells exhibited a decrease in the current signal when exposed to PE particles [276]. This decline in current was directly correlated to the increasing concentration of MPs and was observed over 42 days. The binding of PE particles to the microbial biofilm led to an increase in internal resistance (Rct) within the system, resulting in the observed decrease in the current signal [276].

Due to their ability to adsorb pollutants, it is essential to note that MPs can serve as carriers for other contaminants in the environment. Additionally, they can release harmful plastic monomers and additives/chemicals from their polymeric structure, including polybrominated diphenyl ethers (PBDEs), nonylphenol, bisphenol A (BPA; 4,4′-(propane-2,2-dial)diphenol), and phthalates. In particular, bisphenol A is a potent endocrine disruptor that mimics the behavior of hormones in the endocrine system [244]. In the study conducted by Vidal et al. (2023), BPA was used as a reference pollutant that PS-MPs adsorbed. A differential pulse voltammetry technique was then employed to quantify BPA in MP suspensions, achieving a linear range of 0.80–15.00 µM and a detection limit of 0.24 µM [244].

In 2020, Annamalai and Vasudevan developed the first reported electrochemical biosensor for detecting phthalate esters (PEs) in PET bottles and lake water contaminated with industrial effluents. They conducted a Linear Sweep voltammetry analysis of a Nafion (NF) surface-modified glassy carbon electrode (GCE), which was enhanced with esterase (EST) and nano-components. The peak potential of the individual PEs ranged from −1.72 to −1.82 V at a concentration of 1 × 10^−5^ mM. The sensitivity of the biosensor was determined in terms of the detection limit, which was calibrated to be between 0.03 and 0.08 nM. The results were compared with those obtained from other electrochemical methods for PE detection, demonstrating the effectiveness of the EST and nanocomposite material utilized in the NF-modified GCE for detecting PEs in the selected samples. This biosensor can be further modified and optimized for additional environmental or food studies [277].

Later, the study conducted by Gongi et al. (2022) reported an innovative impedimetric sensor utilizing cyanobacterial extracellular polymeric substances (EPSs) to detect four types of MPs, ranging from 0.1 μm to 1 mm in size. This research emphasized the application of EPSs as a sensitive membrane applied to a gold electrode, analyzed through electrochemical impedance spectroscopy. Remarkably, the sensor demonstrated the capability to detect the four MPs at a low LOD of 10 to 11 M. The authors concluded that additional research is required to gain insights into the biosensor’s performance in a mixture of particles of varying sizes [278].

On the other hand, Baumgarten et al. (2023) developed an electrochemical device by coating the surface of a graphene carbon electrode with guava (*Psidium guajava*) extract. The purpose of this device was to detect BPA in MPs. The resulting biosensor demonstrated the ability to accurately and precisely measure trace amounts of BPA in MP samples. It exhibited significant sensitivity, stability, repeatability, and a detection limit of 15.0 nmol/L. Recovery data ranged from 92 to 109%, and the biosensor’s efficiency was validated through comparison with UV-vis spectrometry [279]. Meanwhile, Zheng et al. (2023) developed a biosensor based on a carbon/rhodamine B nanohorn to detect the cytotoxicity of PS MPs and its combined toxicity with three common pollutants: BPA, pentachlorophenol (PCP), and lead (Pb). The electrochemical biosensor utilized normal human hepatocytes (L-02) as a model for toxicity assessment. The half-inhibitory concentrations (IC50) for PS, BPA, PCP, and Pb in L-02 cells were found to be 286.34 μg/mL, 78.85 μM, 67.87 μM, and 60.12 μM, respectively [280]. Additionally, the study explored oxidative stress and cell apoptosis. The Toxicity Units (TU50) for the combinations of BPA/PS, PCP/PS, and Pb/PS in L-02 cells were 0.97, 0.98, and 1.16 TU, respectively, indicating that PS has additive effects when combined with these three typical pollutants [280].

The use of nanostructures was also employed as sensing enhancements. For example, Shan et al. (2023) developed an electrochemical biosensor for the detection of hydroquinone (HQ) using oriented Prussian blue/polyaniline (PB/PANI) nanoarrays [281]. This biosensor demonstrated reliability in analyzing lake water samples, achieving a high sensitivity of 931.39 mA mM^−1^ cm^−2^ and a LOD of 250 nM (0.027 ppm) after the immobilization of laccase. As a result, this biosensor is highly reproducible and reusable due to its excellent accuracy in lake water analysis. Finally, Zhao et al. (2023) proposed a low-cost electrochemical biosensor for detecting phenolic compounds, specifically catechol (CC) and hydroquinone (HQ), in water samples. Their approach involved the co-immobilization of cell surface-exposed bacterial laccase (CSDBLac). The biosensor demonstrated remarkable sensitivity, with a low LOD of 0.15 mM for CC and 0.09 mM for HQ. It also exhibited excellent repeatability and stability, which can be attributed to the synergistic effects of nanohybrids and CSDBLac [282]. Furthermore, the biosensor did not show significant amperometric responses for other phenolic compounds. However, the authors suggest further exploration to improve the system for simultaneous and differentiated detection of CC and HQ [282].

### 3.2. Plasmonic Sensing Approaches

**Figure 4:**
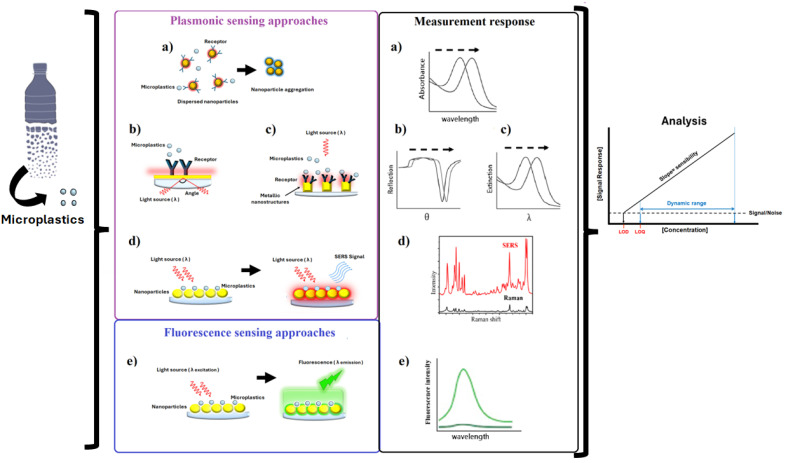
Scheme of plasmonic and fluorescence sensing approaches: (**a**) colorimetric methods, (**b**) surface plasmon resonance, (**c**) localized surface plasmon resonance, (**d**) surface-enhanced Raman spectroscopy, and (**e**) fluorescence for the analysis of microplastics.

The optical properties of metal NPs have been increasingly applied in sensing and analytical applications. When metallic nanoparticles are exposed to incident light, the metal’s conduction electrons become excited, leading to a collective oscillation known as localized surface plasmon [283]. This optical phenomenon can be utilized in various ways to develop different analytical methods for sensing (see Figure 4). These methods include colorimetric approaches, where a color change occurs due to the aggregation of nanoparticles; surface plasmon resonance techniques, in which the reflectance of a sensor chip changes due to the surface plasmon phenomenon; and plasmon-enhanced fluorescence methods, which enhance the emission properties of the nanomaterial [284]. Additionally, surface-enhanced Raman spectroscopy (SERS) is another optical technique that employs nanomaterials. This technique amplifies weak Raman scattering signals by creating hot spots from molecules adsorbed onto nano-metallic nanostructures [285].

Colorimetric methods offer a simple and low-cost approach to sensing, as they do not require complex instrumentation; the color change can be evaluated with the naked eye. The fundamental principle of this technique relies on the aggregation of colloidal metal nanoparticles, which alters the localized surface plasmon resonance (LSPR) absorption peak, resulting in the color change due to the displacement of the maximum absorption wavelength of plasmon [286]. The detection of MPs using colorimetric methods has been demonstrated with gold nanoparticles. For instance, Hong et al. (2022) utilized the ability of PS NPs to inhibit the acetone-induced aggregation of gold nanoparticles [287]. Their study added a mixed dispersion of PS MPs (sizes 380 and 880 mm) to an acetone solution (up to 60% *v*/*v*), as acetone acts as a solvent for PS particles. This solvent prevented the aggregation of gold nanoparticles in the presence of MPs. Consequently, in the absence of PS MPs, the solution is blue, indicating gold nanoparticle aggregation. However, with the addition of PS MPs, the solution retained the characteristic purple/red color of non-aggregated metal nanoparticles [287]. Later, Behera et al. (2023) developed a colorimetric biosensor using gold nanoparticles as probes in immunochromatographic strips to detect PET. The authors identified synthetic peptide sequences that bind to PET and designed three-dimensional structures to optimize binding with PET monomers, such as BHET, MHET, and other polymeric PET ligands. Their study reported binding affinities through a docking process, revealing that the synthetic peptide SP 1 (WPAWKTHPILRM) exhibited a 1.5-fold increase in binding affinity with BHET and MHET compared to the reference PET-anchoring peptide Dermaseptin SI (DSI). Additionally, the authors noted that different nanoparticle structures, such as rods and stars, can enhance signal amplification, thereby increasing the sensitivity of the biosensor [288]. Furthermore, gold nanoparticles with anchored peptides (LCI or TA2) were also utilized to detect MPs in a concentration range of 2.5 to 15 μg/mL. In this study, gold nanoparticles accumulated on the MPs’ surface, causing the solution’s color to shift from red to blue [245].

In terms of biosensing using colorimetric methods, Li et al. (2023) developed sensing probes featuring platinum–gold nanoparticles coupled to antibodies for dimethyl phthalate (DMP) and dibutyl phthalate (DBP). These sensing probes were utilized in a colorimetric immunoassay for DMP and DBP, achieving a linear range of 0.5 to 100 μg/L for DMP, with a detection limit of 0.1 μg/L. For DBP, a linear range of 1 to 32 μg/L was established, with a detection limit of 0.5 μg/L. Compared to traditional immunoassays, such as the ELISA method, this biosensor demonstrated improved detection sensitivity, particularly in real matrices from baijiu and other plastic-bottled beverages [246]. Additionally, other bioreceptors used in colorimetric methods include laccase enzymes coupled to copper nanoparticles with a carbon nitride skeleton and triazole groups (Cu-g-C3N5). These nanocomposites were applied in the colorimetric detection of BPA and exhibited an analytical performance within a linear range of 0.25 to 25 mg/L, with a detection limit of 0.09 mg/L [247]. It is important to note that while colorimetric methods offer simplicity and sensitivity for detecting MPs, several significant challenges remain for their practical application. For instance, no data regarding potential interferents are currently available, and more quantitative analyses are necessary. Most studies primarily report the presence or absence of MPs without establishing critical analytical parameters, such as limits to detection or sensitivities. To improve data interpretation, utilizing spectrophotometric information alongside visual assessment could be beneficial.

Surface plasmon resonance (SPR) sensing is a label-free and non-destructive technique that takes advantage of the plasmon phenomenon occurring on the surface of a thin layer of metal, typically gold or silver. When light excites the free electrons in these metals, it causes collective oscillations [289]. Due to this plasmon resonance effect, the metallic layer’s surface is sensitive to changes in mass, which are indicated by variations in refractive index that affect SPR measurements. Bioreceptors such as enzymes or antibodies are immobilized on the surface to enhance the specificity of detecting target molecules. When binding events occur, they result in conformational changes or mass increases, which can be detected as changes in the intensity of reflected light or shifts in the SPR angle (the angle of maximum absorption wavelength) [290].

Regarding MP detection, Tuoriniemi et al. (2016) proposed an SPR method to detect PS particles with nominal diameters of 100, 300, and 460 nm. The study involved measuring variations in the effective refractive index caused by the absorption of PS nanoparticles on a 50 nm gold film. To validate whether the refractive index changes were proportional to the particle sizes in the three standard colloidal solutions, the authors applied a fitting model based on the coherent scattering theory (CST). They reported that the 100 nm samples exhibited a higher refractive index than predicted by the CST model, while the 300 nm particles showed a smaller diameter than what was detected by SPR in comparison to SEM microscopy [291].

Later, Huang et al. (2021) proposed an SPR biosensor that utilizes a gold chip immobilized with estrogen receptors (ERs) as a selective recognition element for detecting MPs, specifically PS, PVC, and PE. The MP samples were sourced from the Plastics Industry Development Center. They underwent a grinding process using 1200 mesh sandpaper, followed by filtration through a micrometer paper filter, resulting in an average particle size of 20 µm for the study [292]. In their research, they immobilized Erα, one of the two isoforms of ERs, which exhibited the strongest binding interaction with PS particles, indicated by a dissociation constant (Kd) of 0.05 nM. This was followed by PVC with a Kd of 0.09 nM and PE with a Kd of 0.14 nM. Additionally, the authors noted a correlation between the number of particles and the intensity of the SPR response [279]. Similarly, Seggio et al. (2024) developed a biosensor that also immobilizes an ER onto a polymer-based gold nanograting (GNG) plasmonic platform. This biosensor is designed to directly detect and quantify spherical poly(methyl methacrylate) (PMMA) NPs in seawater without the need for any sample pretreatment. It was tested with sample volumes of 2 μL and achieved detection within 3 min, with a detection limit of 0.39 ng/mL [248]. Another example of the use of SPR systems was achieved by Oh et al. (2021), who developed a localized surface plasmon resonance (LSPR) system based on customized gold nanoparticles (Au NPs) with bio-mimicked peptide probes designed to target NPs. By chemically conjugating probes onto both the 40–50 nm Au NPs on the LSPR chip and the intercalated 5 nm Au NPs, they achieved specific targeting through oligopeptide recognition. The use of sandwich binding enhanced the LSPR detection sensitivity by up to 60% due to consecutive plasmonic effects [293]. This biosensor was tested in microwave-boiled deionized water within an expanded PS container to detect potential PS NPs. Furthermore, the binding of various morphological forms of NPs to the LSPR sensor was examined and validated using field emission scanning electron microscopy (FE-SEM) analysis. This biosensor proves to be an efficient tool for straightforward measurement of NPs, regardless of their shape, transparency, and morphology [293].

Plasmon-enhanced fluorescence (PEF) is a well-known technique that enhances the emission of weakly fluorescing molecules when they are near plasmonic nanostructures [285]. A PEF method has been reported for detecting particles and fibers ranging from 0.8 to 2.5 µm made of low-density polyethylene (LDPE), poly(butylene adipate-co-terephthalate) (PBAT), and epoxy resins. In this study, MP samples were suspended in miliQ water, cast on gold nanopillar substrates, and then dried. The samples were further analyzed using fluorescence microscopy [249,294]. The results indicated a LOD of 0.35 femtograms and a LOQ of 1.2 femtograms. These findings demonstrated a significant improvement in MP measurement, with a factor of 68 increase in the signal-to-noise ratio when using the nanopillar structure compared to bare glass. The method also yielded reliable results from sweater samples [249].

Metallic nanoparticles can also be used for analytical purposes when coupled to Raman spectroscopy techniques, resulting in surface-enhanced Raman spectroscopy (SERS). This optical method amplifies the weak Raman scattering signals of molecules adsorbed on metallic nanostructures, producing increased signal intensities [286]. The SERS technique has been widely used to identify the fingerprints of MPs in various samples, enabling the differentiation of a range of polymeric compounds, such as PS, polymethyl-methacrylate, and PET, based on their Raman spectra. For instance, Kihara et al. (2022) developed a filter paper-based method utilizing gold nanoparticles approximately 20 nm in size, achieving a LOD of 1 µg/mL. In their study, the concentration of NaCl was tested to simulate real biological samples or seawater conditions, which reduced detection performance to 100 µg/mL of PS particles. This decrease was attributed to the electrostatic repulsion between the metallic and plastic particles [253]. Subsequently, gold nanorods were investigated for detecting 350 nm PS particles, yielding a comparable result with a LOD of 6.25 µg/mL [295], which is similar to the findings of Kihara et al.

In terms of silver nanoparticles, Lv et al. (2020) described the detection of PS MPs sized 100 and 500 nm, achieving a LOD of 40 µg/mL in seawater [250]. In contrast, Zhou et al. (2021) reported a lower LOD at 5 µg/mL for PS NPs measuring 1 µm and 50 nm [183]. The authors also noted interference peaks during measurements that were attributed to matrix effects from river water samples. Similarly, Hu et al. (2022) investigated PS NPs of sizes 50, 100, 200, and 500 nm, reporting LOD values of 12.5, 6.25, 25, and 25 µg/mL, respectively [252]. Furthermore, the addition of co-adjuvants during the SERS measurements has been shown to enhance the aggregation of metallic nanoparticles, thereby improving the detection of MPs. In this context, Park et al. (2022) reported that the stabilizer cetyl trimethyl ammonium bromide (CTAB) effectively stabilized gold nanorods, allowing for the detection of larger PS particles (0.1, 0.5, and 1 µm), with a lowest LOD of 1 µg/mL specifically for the 0.1 µm particle size [296].

Noticeably, the structure of the metallic nanoparticles significantly affects the performance of SERS methods. For instance, a 3D-crossed gold nanowire enabled the detection of PS particles smaller than 1 µm. Similarly, an inverted pyramid shape with a grid structure featuring cavities facilitated the detection of nanoparticles smaller than 360 nm [297]. Additionally, cellulose hydrogel films containing gold and silver nanowires have been reported to enhance detection [255]. V-shaped nanopores with deposited gold nanoparticles also demonstrated superior detection capabilities compared to spheric metal nanoparticles [298]. More recently, Ahn et al. (2024) reported the design of peptide-decorated microneedles with gold nanorods for the specific detection of polystyrene, polypropylene, and polyethylene microplastic detection [299]. In particular, the peptides present greater stability, compared to antibodies, towards environmental conditions, overcoming some of the limitations of in situ applications. Thus, the design of peptides for the detection of microplastics has gained recent interest in the research community. For example, Woo et al. (2022) tested peptides for polystyrene and polypropylene, evaluating their affinity in deionized water and saline solution to mimic sea-like conditions. Another aspect considered in the study was the effect of some environmental factors on the plastics, such as UV radiation. Thus, the affinity of the peptides was tested on oxidized microplastics (using O_2_ plasma) [300].

### 3.3. Fluorescence Biosensor Approaches

Fluorescence sensors refer to a luminescence method that occurs during the irradiation of a fluorophore label (fluorescent dyes). When excited, the label emits light with unique wavelengths (fluorescence signal), whose intensity is quantified by fluorescence microscopy or microplate readers. Thus, the interaction of the microplastic or their additives, either by direct binding or detecting released compounds, changes the fluorescence intensity (or emission wavelength changes) [77]. This detection scheme has been proposed by Dierkes et al. (2022), who designed an ultrasensitive fluorescent biosensor for MP monitoring. The biosensor enables the rapid detection of degradation products from PET through the enzymatic activity of the Gram-negative bacterium *C. thiooxidans* S23 (DSM17888). The authors discovered that a deletion mutant of *C. thiooxidans* serves as a versatile reporter platform for monitoring terephthalic acid (TPA) concentrations as low as 1 nM. TPA is a monomer that constitutes PET, making this biosensor the most sensitive TPA detection system reported to date. The biosensor construct features the *tphC* promoter, which encodes the protein terephthalate (TA) permease, fused to superfolder green fluorescent protein (sfGFP). This design provides fluorescent readouts after 2 to 4 h of incubation of *C. thiooxidans* [301]. Meanwhile, Puhakka and Santala (2022) developed a method for detecting acrylic acid (AA) monomers using a bioluminescent bacterial biosensor designed for aquatic environmental monitoring. They utilized *Escherichia coli* pBAV1K-ACU-lucFF as the host organism, which incorporates a luciferase-based reporter system under the control of an acrylic acid-specific promoter [302]. The bioluminescent biosensor cells can differentiate degradation products released by AA in saltwater and spiked lake water samples. The fluorescence index (FI) of the *lucFF* sensor is approximately 1.03 at a concentration of 100 μM, which is comparable to the performance of the GFP sensor at 10,000 μM. The highest FI values were observed at AA concentrations of 1000 and 10,000 μM AA. This rapid detection technique shows promise for high-throughput applications and could be adapted to other monomers by redesigning the sensor elements. The authors suggest potential improvements to increase the signal-to-noise ratio, such as optimizing the sensitivity of the transcription factor or enhancing the cells’ tolerance to the toxic effects of AA by transforming the plasmid into a more tolerant bacterial species [302]. Table 3 displays some of the advantages and disadvantages of the novel sensing approaches reviewed for MP and NP detection.

## 4. Prospects and Future Challenges

The detection and monitoring of micro- and nanoplastics in real samples present a complex challenge for the global research community. While several advancements have been made in developing sensing platforms, their successful implementation—especially in environmental matrices rich in impurities such as microorganisms, organic matter, suspended particles, and salt concentrations—remains a significant challenge. These impurities can interfere with measurements, leading to false positives or hindering the analytical performance of the devices. Therefore, a critical aspect of this process is the selection of recognition elements, such as enzymes or antibodies, which ensure the specificity of the method by distinguishing the polymeric material of MPs and NPs from other contaminants in real samples. Antibodies typically offer the highest sensitivities and selectivities but are the most susceptible to environmental factors. Finding receptors (such as enzymes, antibodies, peptide sequences, or even cells) that target plastic particles in nature is very challenging since only a few organisms (some fungi and bacteria) have evolved their mechanisms to produce specific receptors for certain plastic molecules caused by their interaction with the plastic in the ecosystem, for example, the PETase enzyme production by bacteria *Ideonella sakaiensis* [308].

Meanwhile, the design of synthetic molecules with functional groups that can react with plastic particles has gained relevance in research, creating unique nanostructures, nucleotide sequences, peptides, polymers, or fluorescent dyes that bind the plastic target. In particular, the aptamers technology consists of small single-stranded (ss) DNA or RNA ligands, which can be designed to exhibit higher affinity and selectivity toward target analytes, such as polymers and additives present in microplastics [309]. The Systematic Evolution of Ligands by Exponential Enrichment (SELEX) is a process that allows the design of the aptamers by selecting (from vast libraries) oligonucleotide sequences that exhibit target binding affinity. In addition to their quick synthesis, the intricate structure of aptamers (including multi-branched loops and quadruple structures) makes them extremely stable and resistant to harsh environments. In this context, aptamer-based detection methods have been studied to detect microplastic leachates, such as phthalic acid ester, in water samples by fluorescence intensity measurements [310] or bisphenol A (BPA) in aqueous samples using an array of interdigitated microelectrodes by the changes in capacitance caused by the interaction between BPA and the immobilized aptamer [311] or based on a competitive binding scheme with a DNA probe immobilized on a gold electrode [312]. Additionally, the design of peptides that specifically bind to various plastics is gaining relevance as a recognition element in sensing. Peptides are generally less expensive to produce than antibodies, and their design can be optimized to enhance stability, selectivity, and sensitivity through careful amino acid combinations [300].

However, despite these advancements in receptor design, the major challenge is that plastic particles vary significantly in molecular structure even within the same type of polymer by incorporating pigments, stabilizers, flame retardants, etc., or by environmental transformation caused by weather factors (humidity, solar radiation, etc.), leading to plastic’s oxidation and shifting towards a more hydrophilic nature [179], implying a great drawback in the obtention of a universal receptor for microplastic detection.

On the other hand, most research on the detection technologies reviewed has been conducted under laboratory conditions with standard microparticle suspension solutions. Consequently, crucial factors such as MP polymer composition, shapes other than spherical (such as fibers and pellets), and matrix effects have received less attention during the research. These factors are often overlooked and indicate that monitoring is still in the “proof of concept” phase. In this sense, one of the most serious problems of sensing systems under real scenarios is the surface fouling caused by the passive adsorption of foreign materials in the sample (algae, cells, bacteria, and organic matter), which induces a decrease in the analytical performance (sensitivity, resolution, and reliability) and lifetime of the sensor [313]. However, as most of the sensor performance research has been reported in buffer solutions, there is no information on the fouling effect of sensors’ surfaces during microplastic analysis. This suggests several enhancements are needed to transition sensing technology from the lab to real environmental scenarios. Prospects include the development of procedures that may be applied in situ, with or without sample pretreatment steps, allowing for real-time sensing and reporting of the analytical performance of the assay. With this attempt to operate in the field, most of the sensors comprise coupled microfluidic systems that allow in situ sampling and real-time monitoring, with portability capacity, especially electrochemical sensors, which have substantially advanced in miniaturization of their components [314]. However, none has demonstrated operational conditions for field microplastic detection, and only a few have tested the devices in real samples (without determining matrix effects). Also, the advances in electrochemical technology in terms of multiplexing platforms are interesting, allowing the detection of various molecules simultaneously by multiple probes to confer strong qualities to next-generation electrochemical biosensors for their implementation in the field of multi-recognition of polymer MPs. These efforts will contribute to establishing regulatory frameworks and guidelines for plastic regulations.

## 5. Conclusions

This comprehensive review presented the current status of various sensing approaches for monitoring micro- and nanoplastics, which have become prevalent daily. In this context, nanomaterials were emphasized as crucial components in developing (bio)sensing platforms, significantly improving the analytical performance of these devices. However, the recognition elements, such as enzymes or antibodies, are key for achieving the sensitivity and specificity necessary for detecting MPs and NPs in real environmental applications. This study also provided insights into the different transduction principles of (bio)sensors, encompassing the electrochemical and optical detection mechanisms and the various biorecognition elements used, offering valuable information regarding the analytical performance of different sensors, such as the size of MPs detected, limits of detection, and sensitivities, while also discussing measurement conditions, strengths, and weaknesses. Additionally, the review included a section discussing prospects, addressing the challenges and potential outcomes that could lead to significant advancements in environmental monitoring, highlighting the necessity of testing the new sensing developments under real conditions, and considering the composition/matrix of the samples, which are often overlooked. Finally, it is important to address the importance of more studies that address using peptides as a recognition element in microplastic sensing. These receptors have gained relevance in recent years for their unique properties.

## Figures and Tables

**Table 1:** Overview of the advantages and disadvantages of the current detection methods for micro- and nanoplastics.

Method	Advantages	Limitations
Stereomicroscope	Rapid and facile technique. Can identify shape, color, and size.	It is impossible to determine plastic particles’ composition, additives, and nature. There are no available data on transparent and small-size particles. Morphological estimation may lead to miscalculation of microplastic numbers.
SEM	High-resolution images with good clarity. If bound with EDS, elemental analysis is possible. No gas and sputtering when combined with ESEM mode. STEM mode can identify small particles. Sample treatment is not necessary.	Costly equipment. Longer duration for analysis. Composition cannot be identified.
Fluorescence microscope	Easy and immediate visualization of microplastics. A facile strategy for the detection of transparent particles.	Ultraviolet radiation can be toxic and harmful. Chemical additives of the plastic particles can misinterpret the result.
Fourier transform infrared spectrophotometer (FT-IR)	Facile sample preparation and no pretreatment. Chemical composition can be identified. The fingerprint region can reveal the distinction between plastic particles. If ATR is attached, solid, liquid, film, and powder samples can be analyzed. Less than 20 µm size particles can be identified using µ-FTIR Non-destructive method.	Costly equipment. The detection factor is limited by wavelength radiation. Time consumption for every particle identification.
Raman spectroscopy	A technique to identify smaller size microplastics (1 mm). Non-destructive, gaseous films, solids, and single-crystal samples can be analyzed.	It is an expensive and time-consuming technique. Interference may come from pigments and fragments released by adhesive polymers.
Thermal GC-MS	Unknown plastic particles can be identified based on the mass particle.	Number and size information is not detectable.
Inductively coupled plasma–mass spectrometry (ICP-MS)	Several particles’ chemical properties, density, and mass concentration can be identified easily.	It is a costly and destructive technique.
Laser direct infrared spectroscopy (LDIR)	Detect particles up to 20 µm in soil, groundwater, ocean, and biological tissues.	Costly equipment. Smaller size (<20 µm) particles cannot be identified.

**Table 2:** Performance summary of various electrochemical sensors to detect microplastics and their released harmful plastic monomers/additives/chemicals.

Micro- and Nanoplastic Detection				
Method	Analyte	Limit of Detection	Measurement Conditions	Reference
Resistive pulse sensors	Microparticles 21.9 µm	6.52 × 10^−4^ particles/mL	Salt concentrations ranging from 2.5 × 10^−4^ to 0.1 M Sample: teabags	[242]
Impedance spectroscopy Chronoamperometry	Polyethylene particles 212–1000 mm Polystyrene 0.1 to 10 µm	5–500 ng/L	Carbon fiber wire electrode Sample: tap water Carbon electrodes with ferrocene as mediator Sample: water	[243,244].
Colorimetric	Polyethylene terephthalate particles Dimethyl phthalate (DMP) and dibutyl phthalate (DBP) Bisphenol A	2.5–15 mg/L DMP: 0.1 μg/L DBP: 0.5 μg/L 0.09 µg/mL	Gold nanoparticles with anchored peptides Platinum–gold nanoparticles coupled to antibodies for DMP and DBP Sample: baijiu and other plastic-bottled beverages Copper nanoparticles with a carbon nitride skeleton and triazole groups (Cu-g-C3N5) Sample: water	[245,246,247]
Surface plasmon resonance (SPR)	Poly(methyl methacrylate) nanoparticles	0.39 ng/mL	SPR platform with a polymer-based gold nanograting Water Sample: seawater	[248]
Localized surface plasmon resonance (LSPR)	Polystyrene particles	-	Gold nanoparticles (Au NPs) with bio-mimicked peptide probes	[245]
Plasmon-enhanced fluorescence (PEF)	Low-density polyethylene (LDPE), poly(butylene adipate-co-terephthalate) (PBAT), and epoxy resins from 0.8 to 2.5 µm	-	Gold nanopillar substrates Sample: miliQ water	[249]
Surface-enhanced Raman spectroscopy (SERS)	Polystyrene 100, 500 nm PE 10 µm PP 10 µm Polystyrene 1 µm, 50 nm Polystyrene 50 to 2 µm Polystyrene 20 and 200 nm Polyethylene terephthalate 10, 15, 20 µm Polystyrene 84–630 nm Polystyrene (PS) from 50 nm to 1 µm and poly(methyl methacrylate)/PMMA 500 nm Polystyrene (PS), polyethylene terephthalate (PET), polyethylene (PE), polyvinyl chloride (PVC), polypropylene (PP), and polycarbonates (PCs) from 80 to 150 µm	40 µg/mL 5 µg/mL PS 50 nm: 12.5 µg/mL PS 100 nm: 6.25 µg/mL PS 200 nm: 25 µg/mL PS 500 nm: 25 µg/mL PS 1 µm: 12.5–25 µg/mL PS 20 nm: 10 µg/mL PS 200 nm: 1 µg/mL 100 µg/mL PS 84 nm: 100 µg/mL PS 444 nm: 50 µg/mL PS 630 nm: 100 µg/mL PS 84 nm: 500 µg/mL PS 444 nm: 500 µg/mL PS 630 nm: 500 µg/mL PS 50 nm: 10–4 µg/mL PMMA 500 nm: 10–3 µg/mL PS: 1 µg/mL	Silver nanoparticles Sample: seawater Silver nanoparticles Sample: river water Silver nanoparticles Sample: real water Gold nanoparticles Sample: seawater Filter paper with gold nanoparticles Sample: tap water and pond water Silver nanowires on cellulose Gold nanorods on cellulose Silver nanowire array Sample: seafood, market water, and seawater Gold nanoparticle-decorated sponge Sample: seawater, river water, snow water, and rainwater	[250,251,252,253,254,255,256,257]
**Released Harmful Compounds**				
**Method**	**Analyte**	**Limit of Detection**	**Measurement** **Conditions**	**Reference**
Differential pulse voltammetry	Catechol Hydroquinone Hydroquinone Catechol Resorcinol Catechol Hydroquinone Resorcinol Bisphenol A Bisphenol A 4-(methylamino)phenol	0.96 µM 0.56 µM 0.13 µM 0.15 µM 1.36 µM 1.70 µM 5.10 µM 4.50 µM 1.0 µM 0.025 µM 0.0021 µM	Electrode: poly(4-vinylphenylboronic acid)-functionalized polypyrrole/graphene oxide nanosheets Sample: tap water Electrode: cobalt–iron selenides/porous carbon nanofibers/graphene carbon electrode Sample Lake water Electrode: Carbon electrode modified by carbon black/gold sononanoparticle nanocomposite (CB/AuSNPs) Sample: tap, dam, and swamp water Electrode: gold nanoparticles/1,3,5-triformylphloroglucinol and benzidine covalent organic frameworks/graphene carbon electrode Sample: lake water Electrode: metal–organic framework/graphene oxide/carbon paste electrode Sample: lake, tap, and drinking water	[258,259,260,261,262,263]
Square-wave voltammetry	Catechol Hydroquinone Bisphenol A Phenol	0.20 0.16 2.40 3.0	Electrode: silver nanoparticles/multi-walled carbon nanotubes/graphene carbon electrodes Sample: tap water	[264]
Cyclic voltammetry	Catechol 2-aminophenol 2-chlorophenol 2-nitrophenol Catechol	0.045 µM 0.0057 µM 0.0013 µM 0.0010 µM 0.106 µM	Electrode: glutamine-activated graphite paste electrode Sample: Water samples Electrode: CaCu_2_O_3_ nanorod-shaped/graphene carbon electrode Sample: tap water and agricultural water Electrode: sodium dodecyl sulfate modified graphene paste electrode Sample: tap water	[265,266,267]
Colorimetric	Dimethyl phthalate (DMP) and dibutyl phthalate (DBP) Bisphenol A	DMP: 0.1 μg/L DBP: 0.5 μg/L 0.09 mg/L	Platinum–gold nanoparticles coupled to antibodies for DMP and DBP Sample: baijiu and other plastic-bottled beverages Copper nanoparticles with a carbon nitride skeleton and triazole groups (Cu-g-C3N5) Water Sample: water	[246,247]
Localized surface plasmon resonance (LSPR)	Bisphenol A	0.0010 µM	Gold nanoparticle-modified	[268]
Surface plasmon resonance	Bisphenol A	0.0087 µM	molecular imprinted polymers based on monomer ethylene glycol dimethacrylate-*N*-methacryloyl-L-phenylalanine-vinyl imidazole Sample: water Time of response: 5 min	[269]
Surface-enhanced Raman spectroscopy (SERS)	Bisphenol A	0.05 µM	Molecular imprinted polymers based on monomer methacrylic acid Time of response: 20 min	[270]

**Table 3:** Overview of the advantages and disadvantages of novel sensing approaches for micro- and nanoplastics.

Transduction Principle	Sensing Technology	Advantages	Disadvantages	Reference
Electrochemical sensors	Voltammetry	Low-cost production of electrodes and microelectronic circuits. A straightforward read-out and processing device. Multiplexing capability.	Conditions of pH and ionic strength in the sample significantly affect the sensor’s performance. The miniaturization of sensor devices tends to increase the signal-to-noise ratio. The lifetime of electrodes diminishes due to fouling effects. Redox molecules tend to be employed for reaction at the working electrode.	[303]
	Resistive pulse sensor	Allows a high concentration detection. A straightforward read-out and processing device. Suitable for different types of plastics.	The sample’s pH and ionic strength significantly affect the sensor’s performance. Restricted size range.	[304]
	Impedance spectroscopy	A straightforward read-out and processing device. Suitable for different types of plastics.	The sample’s pH and ionic strength significantly affect the sensor’s performance. Sophisticated manufacturing and data processing. Comparatively poor recovery rate.	[304]
Plasmonic sensors	SPR	Instruments and chips are already well established in the market (mainly for biological assays). Allows label-free detection schemes (i.e., no addition of fluorescent tags). Highly sensitive to the refractive index and allows multiplexing detection.	The prism is a drawback in miniaturization attempts. Detects refractive index changes close to the metal film surface (extending up to 200 nm). Temperature control is needed to produce stable SPR signals.	[305]
	LSPR	Multiplexing and miniaturization capability. Tuning detection by varying the nanoparticles’ size, shape, and composition. Use of different wavelengths that do not overlap with the spectral of natural chromophores in the samples.	The sensors are susceptible to the refractive index of the surrounding medium. The experiments need to ensure that the binding of the target molecule happens within the sensing volume when it involves bulky molecules.	[306]
	Plasmon-enhanced fluorescence	Present good signal-noise ratio. Damage of sensing elements due to prolonged exposure to incident light. Allows discrimination of plastics with different sizes and different compositions.	Complex instrumentation. No studies have been reported in real samples for microplastic detection. Slow response time due to the diffusion effect of analytes.	[307]
	Colorimetric methods	The plasmon resonant nanostructures can be used as fluorophore tags. Easy to use. No expensive instrumentation required. Allows fast qualitative screening tests.	Unable to provide reliable quantitative measurements.	[307]
Surface-enhanced Raman spectroscopy		Provides simultaneous quantitative and qualitative detection by combining the Raman fingerprints of different analytes and plasmonic nanostructures of the systems (required for the differentiation of MP size and composition). Allows real-time data processing by the combination of fiber optics and microfluidic circuits.	Lack of a standard methodology for sample preparation and a standardized procedure of analysis. It is challenging to perform accurate detection in the field. No matrix effects have been established.	[44]

## Data Availability

The data presented in this study are available upon request from the corresponding author.
